# The Effect of Sitagliptin on the Regression of Carotid Intima-Media Thickening in Patients with Type 2 Diabetes Mellitus: A Post Hoc Analysis of the Sitagliptin Preventive Study of Intima-Media Thickness Evaluation

**DOI:** 10.1155/2017/1925305

**Published:** 2017-01-17

**Authors:** Tomoya Mita, Naoto Katakami, Toshihiko Shiraiwa, Hidenori Yoshii, Masahiko Gosho, Iichiro Shimomura, Hirotaka Watada

**Affiliations:** ^1^Department of Metabolism and Endocrinology, Juntendo University Graduate School of Medicine, Hongo 2-1-1, Bunkyo-ku, Tokyo 113-8421, Japan; ^2^Department of Metabolic Medicine, Osaka University Graduate School of Medicine, 2-2 Yamadaoka, Suita, Osaka 565-0871, Japan; ^3^Department of Metabolism & Atherosclerosis, Osaka University Graduate School of Medicine, 2-2 Yamadaoka, Suita, Osaka 565-0871, Japan; ^4^Shiraiwa Medical Clinic, 4-10-24 Houzenji, Kashiwara, Osaka 582-0005, Japan; ^5^Department of Medicine, Diabetology & Endocrinology, Juntendo Tokyo Koto Geriatric Medical Center, Shinsuna 3-3-20, Koto-ku, Tokyo 136-0075, Japan; ^6^Department of Clinical Trial and Clinical Epidemiology, Faculty of Medicine, University of Tsukuba, 1-1-1 Tennodai, Tsukuba, Ibaraki 305-8575, Japan

## Abstract

*Background.* The effect of dipeptidyl peptidase-4 (DPP-4) inhibitors on the regression of carotid IMT remains largely unknown. The present study aimed to clarify whether sitagliptin, DPP-4 inhibitor, could regress carotid intima-media thickness (IMT) in insulin-treated patients with type 2 diabetes mellitus (T2DM). *Methods*. This is an exploratory analysis of a randomized trial in which we investigated the effect of sitagliptin on the progression of carotid IMT in insulin-treated patients with T2DM. Here, we compared the efficacy of sitagliptin treatment on the number of patients who showed regression of carotid IMT of ≥0.10 mm in a post hoc analysis. *Results*. The percentages of the number of the patients who showed regression of mean-IMT-CCA (28.9% in the sitagliptin group versus 16.4% in the conventional group, *P* = 0.022) and left max-IMT-CCA (43.0% in the sitagliptin group versus 26.2% in the conventional group, *P* = 0.007), but not right max-IMT-CCA, were higher in the sitagliptin treatment group compared with those in the non-DPP-4 inhibitor treatment group. In multiple logistic regression analysis, sitagliptin treatment significantly achieved higher target attainment of mean-IMT-CCA ≥0.10 mm and right and left max-IMT-CCA ≥0.10 mm compared to conventional treatment. *Conclusions*. Our data suggested that DPP-4 inhibitors were associated with the regression of carotid atherosclerosis in insulin-treated T2DM patients. This study has been registered with the University Hospital Medical Information Network Clinical Trials Registry (UMIN000007396).

## 1. Background

Type 2 diabetes mellitus (T2DM) is a risk factor for cardiovascular disease (CVD). Recent studies have cast doubt on the benefits of strict glycemic control on CVD in patients with advanced atherosclerosis or longstanding T2DM [1–3]. Particularly, strict glycemic control using intensive insulin therapy increases the risk of hypoglycemia [4] and weight gain [5] which might reduce their beneficial effects. Therefore, a reduction in insulin dose by stimulation of endogenous glucose responsive insulin secretion and increased insulin sensitivity using oral hypoglycemic agents (OHA) may reduce these adverse effects of insulin therapy and be a promising strategy to prevent the progression of atherosclerosis.

The carotid artery intima-media thickness (IMT) and its progression are considered a marker of progression of atherosclerosis. Recent studies demonstrated that add-on therapy of metformin [6] or pioglitazone [7] to insulin therapy did not slow down the progression of carotid IMT compared with control group in patients with T2DM. On the other hand, we recently reported that treatment with sitagliptin, a dipeptidyl peptidase-4 (DPP-4) inhibitor, attenuated the progression of carotid IMT in insulin-treated patients with T2DM compared with the conventional treatment without increasing the risk of hypoglycemia [8].

Previous studies demonstrated that several therapies for cardiovascular risk factors including statin could regress carotid IMT in patients with T2DM [9–12]. However, the effect of DPP-4 inhibitors on the regression of carotid IMT remains largely unknown. In this study, we investigated whether sitagliptin has beneficial effect on regressing carotid IMT in a post hoc analysis while the original article focused on investigating the effects of sitagliptin on IMT progression.

## 2. Methods

### 2.1. Study Population

We performed a post hoc analysis from the Sitagliptin Preventive Study of Intima-Media Thickness Evaluation (SPIKE). The study design, inclusion and exclusion criteria, study schedule, and measurements were described in detail previously [8]. Briefly, insulin-treated Japanese T2DM patients, free of past history of apparent CVD, who periodically attended the outpatient diabetes clinics at 12 centers across Japan were asked to participate in this study. Randomization was performed using a dynamic allocation method based on the number of times of insulin injection, with/without pioglitazone, age, and gender. A total of 282 participants were randomly allocated to either the sitagliptin group (*n* = 142) or the conventional treatment group (using drugs other than the DPP-4 inhibitor) (*n* = 140). Finally, 137 in the sitagliptin group and 137 in the conventional treatment group were included in the full analysis set. Each participant underwent ultrasonography of the carotid arteries performed by expert sonographers at the start of the study, and the procedure was repeated after 52 and 104 weeks as reported previously [8]. To avoid interreader variability, all scans were electronically stored and emailed to the central office (IMT Evaluation Committee, Osaka, Japan) to be read by a single experienced reader blinded to the clinical characteristics of the patients, in a random order, using automated digital edge-detection software (Intimascope; MediaCross, Tokyo, Japan). The software system averages about 200 points of IMT values in the segment 2 cm proximal to the dilation of the carotid bulb (mean-IMT-CCA). The greatest thicknesses of IMT, including plaque lesions in the common carotid arteries (max-IMT-CCA), were also measured separately. The reproducibility of IMT measurement was very high as described previously [8]. Carotid IMT regression was defined as a decrease of ≥0.10 mm in mean-IMT-CCA, right max-IMT-CCA, and left max-IMT-CCA at the end of the study from the baseline.

### 2.2. Statistical Analysis

Data were reported as mean ± standard deviation. The number and percentage of patients who achieved the regression of IMT ≥0.10 mm from the baseline at the end of the study regression were presented using Fisher's exact test. Multiple logistic regression analysis was performed to compare target attainment of IMT ≥0.10 mm from the baseline at the end of the study between the two groups. Classical atherosclerotic risk factors based on clinical judgment were included in the model. Adjusted odds ratio (OR) estimates and Wald 95% confidence interval (CI) were calculated. Multivariate logistic regression models were used to identify the factors for regression of IMT ≥0.10 mm from the baseline at the end of the study. Classical atherosclerotic risk factors and changes in some of them based on clinical judgment were included in the model. All statistical tests were two-sided with 5% significance level. All analyses were performed using the SAS software version 9.4 (SAS Institute, Cary, NC).

## 3. Results

### 3.1. Baseline Clinical Characteristics and the Results of the Original Article

**Figure 1:**
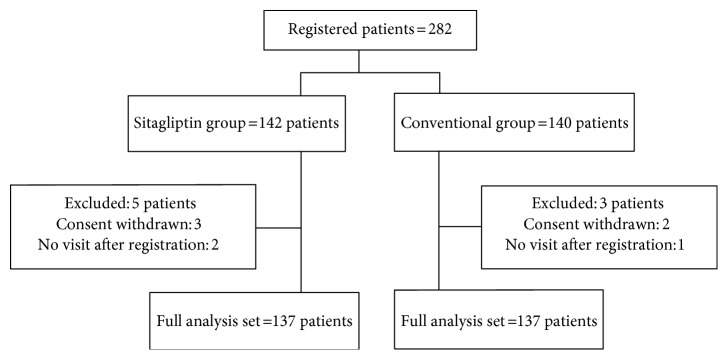
Trial schema.

As described previously [8], baseline clinical characteristics including potential risk factors for carotid atherosclerosis were comparable between the groups (Table 1). The mean age was about 64 years, about 60% male, and HbA1c was about 8% and the estimated diabetes duration was about 17 years. Lipids and blood pressure of study subjects were relatively well controlled. A total of 282 participants were randomly allocated to either the sitagliptin group (*n* = 142) or the conventional treatment group (using drugs other than the DPP-4 inhibitor) (*n* = 140). Finally, 137 in the sitagliptin group and 137 in the conventional treatment group were included in the full analysis set (Figure 1). Changes in the mean (−0.053 mm (−0.090, −0.016) (mean change; 95% CI), *P* = 0.005, by one-sample *t*-test based on mixed effects model for repeated measures) and left maximum IMT (−0.087 mm (−0.161, −0.014), *P* = 0.021), but not right maximum IMT (−0.033 mm (−0.121, 0.054), *P* = 0.45), of the common carotid arteries were significantly greater after sitagliptin treatment compared with conventional treatment (Table 2). Also, the improvement in HbA1c was significantly greater in the sitagliptin group than in the conventional group (Table 2). On the other hand, there were no differences between the two groups in other risk factors such as BP, the lipid parameters at baseline, or their changes during the observation period (Table 2).

### 3.2. The Percentage of Patients Achieving IMT Regression

In this study, we compared the percentage of patients who achieved a decrease of ≥0.10 mm in mean-IMT-CCA and right and left max-IMT-CCA at the end of the study. The percentages of patients with regression of mean-IMT-CCA (28.9% in the sitagliptin group versus 16.4% in the conventional group, *P* = 0.022) and left max-IMT-CCA (43.0% in the sitagliptin group versus 26.2% in the conventional group, *P* = 0.007) were higher in the sitagliptin treatment group compared with those in the conventional treatment group. Also, the percentage in the right max-IMT-CCA tended to be higher in the sitagliptin treatment group compared with that in the conventional treatment group (38.3% in the sitagliptin group versus 27.9% in the conventional group, *P* = 0.10). Multiple logistic regression analysis that included the treatment group, age, gender, and baseline IMT (model 1) demonstrated that sitagliptin treatment significantly achieved higher target attainment of mean-IMT ≥0.10 mm and right and left max-IMT-CCA ≥0.10 mm compared to conventional treatment (Table 3). Similar findings were noted even in the adjusted models, including model 2 (model 1 + body mass index + current smoking); model 3 (model 2 + HbA1c, total cholesterol, high-density lipoprotein cholesterol, triglyceride, and systolic blood pressure); model 4 (model 3 + estimated glomerular filtration rate + angiotensin-converting enzyme/angiotensin II receptor blocker, statin, and antiplatelets); and model 5 (model 4 + OHA).

### 3.3. Predictive Factors for Regression of IMT

Next, multivariate logistic regression models were performed to identify predictive factors for regression of mean-IMT-CCA and right and left max-IMT-CCA. Sitagliptin treatment and higher IMT at baseline were mainly associated with the regression of carotid IMT (Table 4).

## 4. Discussion

Previous study demonstrated that aggressive lipid-lowering therapies with a statin alone or statin and ezetimibe resulted in regression of carotid IMT in patients with T2DM [9]. Theoretically, statin reversed carotid atherosclerosis through reductions in the lipid, inflammation and oxidative stress, and changes in tissue characteristic of plaque. With respect to OHA, previous studies showed that pioglitazone treatment led to the regression of carotid IMT in patient with T2DM probably through a multitude of mechanisms, including improvement in several metabolic factors and insulin sensitivity, anti-inflammatory effect, and direct actions on the vascular cells [10, 11]. In addition, modulation in postprandial hyperglycemia with repaglinide was reported to be associated with the reduction in carotid IMT in patients with T2DM [12]. However, ongoing background treatments with those drugs at baseline, not add-on therapies, did not have major impact on the regression of carotid IMT in this study (Table 4). The effects of these drugs may have already attributed to reduced carotid IMT although we did not collect the data about the treatment periods. Furthermore, sitagliptin treatment was still associated with and was an independent predictive factor for the regression of carotid IMT even considering several possible risk factors for atherosclerosis, background therapies which may have antiatherosclerotic effects, and/or changes in metabolic parameters. Although the exact mechanism by which DPP-4 inhibitors induce the regression of carotid IMT remains uncertain at present, the differences in carotid IMT progression could not be explained by the difference in HbA1c as we discussed in the original article [8]. The glucagon-like peptide-1 dependent and/or independent effect of DPP-4 inhibitors on the vascular wall may attribute to reduced atherosclerosis [13–16].

There are several certain limitations. First, because the limit of detection of IMT measured by ultrasound scanner used in this study was <0.1 mm, we defined a decrease of ≥0.10 mm in IMT as IMT regression in terms of measurement sensitivity in these exploratory post hoc analyses. There are very few studies to investigate the effect of drugs on IMT regression. In a previous study, IMT regression was defined as a decrease of >0.020 mm in mean IMT without reasonable scientific grounds [12]. Whether these magnitudes of IMT regression are clinically relevant still remained unclear. Thus, it may be important to identify the magnitude of IMT regression that is associated with the incidence of CVD. In this regard, we are conducting a study to follow up the incidence of CVD in the same cohort. We will quantitatively assess the relationship between the incidence of CVD and IMT regression. Second, these were exploratory analyses with the concern of multiplicity of statistical testing. These factors allow increased risk for false-negative and false-positive significance results. Thus, the findings should be interpreted with caution.

## 5. Conclusions

In conclusion, our data suggested that DPP-4 inhibitors were associated with the regression of carotid atherosclerosis in insulin-treated T2DM patients free of apparent CVD.

## Figures and Tables

**Table 1:** Clinical characteristics of patients of the two groups.

Parameters	Sitagliptin group (*n* = 137)	Conventional group (*n* = 137)	*P* value
Age (years)	63.8 ± 9.7	63.6 ± 1.0	0.90
Gender (males) (%)	83 (61)	82 (60)	1.00
Body mass index	25.0 ± 4.3	25.0 ± 3.8	0.88
Current smoking	30 (22)	29 (21)	0.22
Duration of diabetes (years)	17.2 ± 8.5	17.3 ± 8.7	0.94
HbA1c at baseline (mmol/mol)	64.9 ± 11.9	63.9 ± 10.6	0.45
Systolic blood pressure (mmHg)	130 ± 16	132 ± 14	0.88
Total cholesterol at baseline (mmol/l)	5.02 ± 0.91	4.94 ± 0.86	0.50
HDL cholesterol at baseline (mmol/l)	1.46 ± 0.37	1.39 ± 0.38	0.14
Triglyceride at baseline (mmol/l)	1.13 (0.83, 1.55)	1.17 (0.90, 1.72)	0.22
eGFR (ml/min/1.73 m^2^)	77.7 ± 21.2	79.7 ± 24.2	0.47
Mean IMT (mm)	0.84 ± 0.19	0.84 ± 0.21	0.81
Right maximum IMT (mm)	1.04 ± 0.29	1.06 ± 0.40	0.69
Left maximum IMT (mm)	1.10 ± 0.32	1.11 ± 0.41	0.87
Use of oral glucose-lowering agents			
Metformin	49 (36)	48 (35)	1.00
Sulfonylurea	17 (12)	15 (11)	0.85
Glinides	2 (1)	19 (14)	<0.001
Thiazolidinediones	13 (9)	11 (8)	0.83
*α*-Glucosidase inhibitor	41 (30)	42 (31)	1.00
Others			
Angiotensin-converting enzyme inhibitors	8 (6)	4 (3)	0.59
Angiotensin II receptor blockers	53 (39)	69 (50)	0.07
Statins	66 (48)	63 (46)	0.81
Antiplatelet agents	29 (21)	30 (22)	1.00

Data are number (%) of patients or mean ± SD values.

IMT, intima-media thickness; eGFR, glomerular filtration rate.

**Table 2:** Effects of sitagliptin on glucose metabolism, blood pressure lipid metabolism, and IMT.

Parameters	Sitagliptin group (*n* = 137)	Conventional group (*n* = 137)	*P* value
HbA1c at baseline (mmol/mol)			
104 weeks (change from baseline)	−5.6 ± 11.4	−2.2 ± 10.0	0.004
Systolic blood pressure (mmHg)			
104 weeks (change from baseline)	0 ± 19	3 ± 17	0.20
Total cholesterol at baseline (mmol/l)			
104 weeks (% change from baseline)	−2.7 ± 15.6	−1.8 ± 14.6	0.63
HDL cholesterol at baseline (mmol/l)			
104 weeks (% change from baseline)	0.2 ± 15.2	−0.5 ± 14.7	0.74
Triglyceride at baseline (mmol/l)			
104 weeks (% change from baseline)	0.0 (−25.1, 44.6)	−1.6 (−24.6, 16.7)	0.35
Mean IMT (mm)			
104 weeks (change from baseline)	−0.03 ± 0.17	0.02 ± 0.14	0.008
Right maximum IMT			
104 weeks (change from baseline)	0.00 ± 0.35	0.02 ± 0.37	0.67
Left maximum IMT			
104 weeks (change from baseline)	−0.06 ± 0.34	0.02 ± 0.29	0.033

Data are mean ± SD or median (range) values. Differences in parameters from baseline to 104 weeks between groups were analyzed by the Student *t*-test or Wilcoxon's rank sum test. IMT, intima-media thickness.

**Table 3:** Results of adjustment randomized comparisons.

	Adjusted odds ratio (95% CI)	*P* value
Mean intima-media thickness		
Model 1	2.29 (1.17–4.47)	0.015
Model 2	2.27 (1.16–4.44)	0.016
Model 3	2.48 (1.24–4.93)	0.010
Model 4	2.60 (1.29–5.28)	0.008
Model 5	2.89 (1.34–6.24)	0.007
Right maximum intima-media thickness		
Model 1	1.77 (1.00–3.13)	0.049
Model 2	1.78 (1.00–3.14)	0.049
Model 3	1.87 (1.04–3.34)	0.036
Model 4	1.88 (1.05–3.40)	0.035
Model 5	2.15 (1.12–4.14)	0.022
Left maximum intima-media thickness		
Model 1	2.22 (1.25–3.92)	0.006
Model 2	2.24 (1.26–3.98)	0.006
Model 3	2.33 (1.28–4.22)	0.006
Model 4	2.43 (1.32–4.48)	0.004
Model 5	2.46 (1.28–4.73)	0.007

Multiple logistic regression analysis included the treatment group, age, gender, and baseline IMT (model 1); model 1 plus body mass index and current smoking (model 2); model 2 plus HbA1c, total cholesterol, high-density lipoprotein cholesterol, triglyceride, and systolic blood pressure (model 3); model 3 plus eGFR, use of angiotensin-converting enzyme/angiotensin II receptor blocker, use of statin, and use of antiplatelets (model 4); model 4 plus the use of oral hypoglycemic agents (model 5).

**Table 4:** Results of multivariate logistic regression models for the regression of IMT of ≥0.10 mm from baseline at the end of the study.

Factor	Mean IMT		Right max IMT		Left max IMT	
	Adjusted odds ratio (95% CI)	*P* value	Adjusted odds ratio (95% CI)	*P* value	Adjusted odds ratio (95% CI)	*P* value
Age (1 year)	1.01 (0.95–1.06)	0.83	0.98 (0.94–1.02)	0.37	1.01 (0.97–1.06)	0.61
Gender (male/female)	1.99 (0.83–4.79)	0.12	0.67 (0.33–1.35)	0.26	1.02 (0.49–2.14)	0.95
Body mass index (1 kg/m^2^)	1.07 (0.95–1.20)	0.26	1.03 (0.94–1.13)	0.56	1.00 (0.91–1.10)	0.98
Estimated duration of diabetes (1 year)	1.02 (0.97–1.07)	0.42	0.99 (0.95–1.03)	0.65	1.02 (0.98–1.07)	0.26
Smoking (yes/no)	1.93 (0.67–5.52)	0.22	0.72 (0.30–1.74)	0.47	1.83 (0.77–4.35)	0.17
HbA1c (1 mmol/l)	1.04 (0.69–1.56)	0.87	1.07 (0.76–1.51)	0.68	1.00 (0.70–1.44)	0.99
Systolic BP (1 mmHg)	0.99 (0.96–1.02)	0.40	1.03 (1.01–1.06)	0.012	0.97 (0.94–0.99)	0.02
Total cholesterol (1 mmol/l)	0.64 (0.35–1.18)	0.16	1.11 (0.69–1.79)	0.68	1.19 (0.72–1.95)	0.49
HDL cholesterol (1 mmol/l)	0.44 (0.11–1.78)	0.25	1.19 (0.38–3.75)	0.76	0.69 (0.22–2.15)	0.52
Triglyceride (1 mmol/l)	0.92 (0.58–1.44)	0.71	0.87 (0.59–1.29)	0.5	1.00 (0.72–1.38)	1.00
eGFR (1 ml/min/1.73 m^2^)	1.01 (0.99–1.03)	0.27	1.00 (0.98–1.01)	0.74	1.01 (0.99–1.03)	0.22
Baseline cIMT (0.01 mm)	1.05 (1.03–1.08)	<0.001	1.02 (1.01–1.03)	<0.001	1.02 (1.01–1.03)	<0.001
Treatment group (sitagliptin treatment/conventional treatment)	3.58 (1.60–7.99)	0.002	2.02 (1.07–3.84)	0.031	2.38 (1.23–4.58)	0.01
ACE/ARB (yes/no)	1.10 (0.50–2.45)	0.81	0.86 (0.45–1.68)	0.67	1.31 (0.66–2.63)	0.44
Statins (yes/no)	0.76 (0.33–1.74)	0.51	1.27 (0.64–2.49)	0.49	0.88 (0.44–1.76)	0.72
Antiplatelets (yes/no)	0.34 (0.12–0.96)	0.04	0.79 (0.35–1.79)	0.57	0.40 (0.17–0.97)	0.042
OHA (yes/no)	0.55 (0.26–1.18)	0.12	0.83 (0.43–1.58)	0.56	0.53 (0.27–1.04)	0.064
Changes in systolic BP at 104 weeks from baseline (1 mmHg)	1.00 (0.98–1.03)	0.94	1.02 (1.00–1.04)	0.079	0.99 (0.97–1.01)	0.45
Changes in HbA1c at 104 weeks from baseline (1 mmol/l)	1.25 (0.84–1.87)	0.27	1.13 (0.80–1.60)	0.49	0.91 (0.64–1.29)	0.59
Changes in total cholesterol at 104 weeks from baseline (1 mmol/l)	0.97 (0.94–1.00)	0.051	0.99 (0.97–1.02)	0.45	0.99 (0.96–1.01)	0.30
Changes in HDL cholesterol at 104 weeks from baseline (1 mmol/l)	1.00 (0.97–1.02)	0.76	1.01 (0.99–1.03)	0.33	1.01 (0.98–1.03)	0.59
Changes in triglyceride at 104 weeks from baseline (1 mmol/l)	1.00 (0.99–1.00)	0.39	1.00 (1.00–1.01)	0.22	1.00 (0.99–1.01)	0.85

Multivariate logistic regression models were used to identify the determination of the regression of IMT of ≥0.10 mm from baseline at the end of the study.

ACE/ARB, angiotensin-converting enzyme/angiotensin II receptor blocker; BP, blood pressure; IMT, intima-media thickness; eGFR, glomerular filtration rate; OHA, oral hypoglycemic agents.

## Abbreviations

CI: Confidence interval
CVD: Cardiovascular disease
DPP-4: Dipeptidyl peptidase-4
IMT: Intima-media thickness
Max-IMT-CCA: Maximum IMT of the common carotid artery
Mean-IMT-CCA: Mean IMT of the common carotid artery
OHA: Oral hypoglycemic agents
OR: Odds ratio
SPIKE: Sitagliptin Preventive Study of Intima-Media Thickness Evaluation
T2DM: Type 2 diabetes mellitus.
